# Hypothalamic GABAergic neurons: their roles in health and metabolic diseases

**DOI:** 10.3389/fendo.2025.1551741

**Published:** 2025-03-10

**Authors:** Bingwei Wang, Yang Yu, Juan Li, Yu Xiong, Xin Zhang, Ying Wan, Ruimao Zheng, Chunxiang Zhang

**Affiliations:** ^1^ Basic Medicine Research Innovation Center for Cardiometabolic Diseases, Ministry of Education, Southwest Medical University, Luzhou, Sichuan, China; ^2^ Department of Cardiology, The Affiliated Hospital of Southwest Medical University, Southwest Medical University, Luzhou, Sichuan, China; ^3^ Nucleic Acid Medicine, Key Laboratory of Luzhou, Southwest Medical University, Luzhou, Sichuan, China; ^4^ Key Laboratory of Medical Electrophysiology, Ministry of Education, Southwest Medical University, Luzhou, Sichuan, China; ^5^ Institute of Cardiovascular Research, Southwest Medical University, Luzhou, Sichuan, China; ^6^ School of Basic Medical Sciences, Southwest Medical University, Luzhou, Sichuan, China; ^7^ Department of Anatomy, Histology and Embryology, School of Basic Medical Sciences, Peking University, Beijing, China; ^8^ Neuroscience Research Institute, Peking University, Beijing, China; ^9^ Key Laboratory for Neuroscience, Ministry of Education/National Health Commission, Peking University, Beijing, China; ^10^ Beijing Life Science Academy, Beijing, China

**Keywords:** hypothalamus GABAergic neurons, metabolism regulation, energy balance, neural projections, neural and molecular mechanisms, obesity

## Abstract

Hypothalamic GABAergic neurons are important in regulating metabolic homeostasis and energy balance. Serving as critical integrators of catabolic and anabolic processes, these neurons orchestrate a broad spectrum of metabolic functions, including feeding, nutrient metabolism, fluid homeostasis, basal metabolism, thermoregulation, and circadian rhythms. Recent advances in neuroscience have facilitated a deeper exploration of the role of hypothalamic GABAergic neurons in metabolic regulation. Emerging research has uncovered key mechanisms through which these neurons modulate energy balance and maintain metabolic balance. These findings not only enhance our understanding of obesity and related metabolic disorders but also underscore the link between hypothalamic dysfunction and prevalent metabolic diseases such as obesity and type 2 diabetes. This review summarizes the latest advancements in our understanding of the role of hypothalamic GABAergic neurons in metabolic regulation. It aims to elucidate the neural and molecular mechanisms underlying hypothalamic control of metabolism, offering new perspectives for the diagnosis and treatment of metabolic disorders.

## 1 Introduction

The hypothalamus is a crucial brain region for regulating metabolic homeostasis and energy balance, orchestrating core functions in nutrient metabolism, appetite control, hydration, basal metabolism, thermoregulation, and physiological rhythms (1–4). These functions are essential for maintaining the equilibrium between catabolic and anabolic processes (5, 6).

## 2 Anatomical location, subdivision, and major nuclei of the hypothalamus

Anatomically, the hypothalamus lies directly above the pituitary gland, surrounds both upper and lower lateral walls of the third ventricle, and constitutes about 0.3% of the total brain volume in humans (3). It is divided into four distinct areas: the preoptic area (anterior to the optic chiasm, marked by arx/nfix); the supraoptic region (above the optic chiasm, with markers rgs16/reln); the tuberal region (within and above the gray tubercle, marked by tbx3/hdc); and the mammillary region (within and above the mammillary bodies, indicated by foxb1) (7).

**Figure 1:**
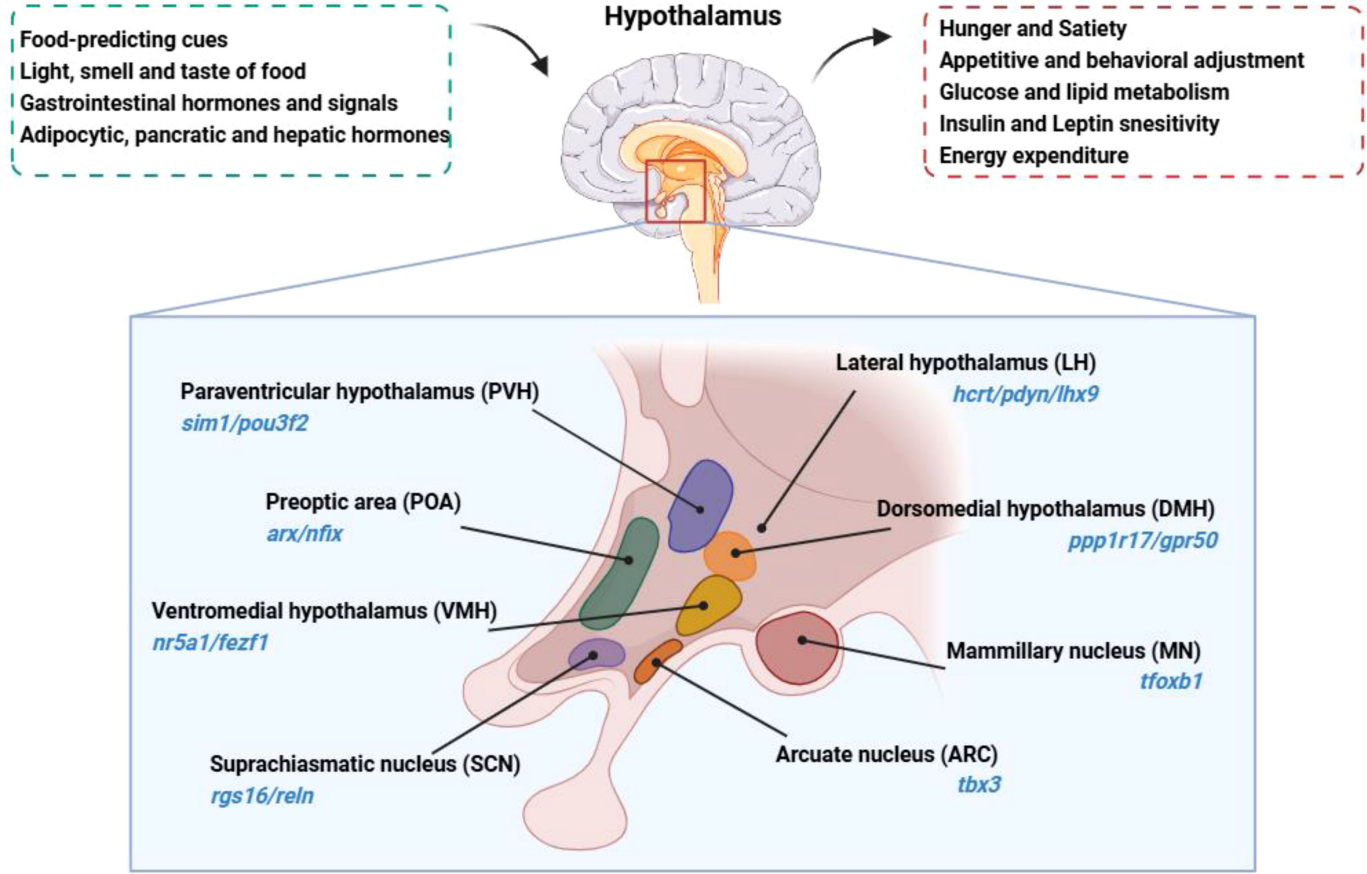
Anatomical location, primary nuclei and function of the hypothalamus is located beneath the dorsal thalamus, surrounds both upper and lower lateral walls of the third ventricle. The hypothalamus senses and integrates signals related to the body’s nutritional and metabolic status, regulating feeding behavior and nutrient metabolism. Hypothalamus nuclei are identified with their respective characteristic molecular markers.

Within this complex structure, hypothalamic regions such as the arcuate nucleus (ARC, marked by tbx3), the paraventricular hypothalamus (PVH, characterized by sim1/pou3f2), the ventromedial hypothalamus (VMH, with markers nr5a1/fezf1), the dorsomedial hypothalamus (DMH, identified by ppp1r17/gpr50), and the lateral hypothalamic area (LH, denoted by hcrt/pdyn/lhx9) play important roles in metabolic regulation (7). These nuclei form integral parts of the neural circuits that underlie various aspects of metabolic control. The anatomical location, primary nuclei and function of the hypothalamus are summarized in **Figure 1**.

GABAergic neurons, which primarily utilize gamma-aminobutyric acid (GABA) as their neurotransmitter, are the principal inhibitory neurotransmitter in the brain (8–10). They are crucial for maintaining the functional balance of the nervous system and suppressing overly excitable states. In the hypothalamus, GABAergic neurons are widely distributed and involved in diverse physiological function and neuroregulatory processes (11). Typically, GABAergic neurons contain glutamate decarboxylase (GAD), an enzyme essential for converting glutamate into GABA (12). Despite their varied morphology, size, and electrophysiological properties, GABAergic neurons share the common function of modulating other neuronal activity through GABAergic signaling (11, 13). By synthesizing and releasing GABA, GABAergic neurons reduce the activity of target neurons through binding to GABA receptors, exerting a potent inhibitory effect (11). This modulation is crucial for maintaining the stability and coordinated function of neural networks (14).

The hypothalamus acts as a critical endocrine and neuroregulatory center, responsible for regulating numerous physiological processes like thermoregulation, hunger and satiety, sleep-wake cycles, and sexual behavior (15–18). GABAergic neurons exist in various hypothalamic areas, including the preoptic area (POA), supramammillary nucleus (SMN), ARC, and DMH. They achieve their effects by inhibiting other hypothalamic neurons and interacting with different neuron types, such as pro-opiomelanocortin (POMC) neurons, agouti-related peptide (AgRP) neurons, and cholinergic neurons (19–21). These interactions are crucial for cooperatively maintaining metabolic homeostasis and energy balance.

Studies have revealed that while GAD-65 and GAD-67 mRNAs are undetectable in the ventromedial hypothalamus (VMH), this region still exhibits GAD activity. This observation suggests that GABA synthesis in the VMH may be primarily associated with GABAergic axon terminals, or possibly via a novel mechanism that has not yet been identified (22). Furthermore, high DLX expression is considered a hallmark of maturation of GABAergic neurons. For example. in *DLX5/6-GFP* mice, a sparse population of GABAergic neuronal cell bodies has been observed within the VMH, indicating that GABAergic neuron may be present in VMH (22). Furthermore, a recent single-cell sequencing study also identified GAD-67 positive neurons in the VMH (23). Although these findings suggest that GABAergic neurons may exist in the VMH, further research is needed to determine their exact prevalence and functional significance in metabolic regulation, which may ultimately provide novel insights into how this critical brain region influences overall physiological homeostasis.

In summary, GABAergic neurons in the hypothalamus are crucial for maintaining physiological balance and neural stability. They are distributed across multiple hypothalamic areas and participate in regulating various physiological processes through inhibitory neurotransmission. Dysfunction in the metabolic regulatory roles of hypothalamic GABAergic neurons may lead to metabolic disorders, such as obesity, type 2 diabetes, and metabolic syndrome. With the global surge in obesity and related metabolic diseases causing significant health problems and societal concerns, the exploration of the mechanisms by which hypothalamic GABAergic neurons regulate metabolism and energy balance is not only crucial for understanding the pathogenesis of these diseases but also for identifying effective new targets for their treatment.

## 3 Hypothalamic GABAergic neuronal nuclei: central players in metabolism and energy balance

In the ARC, GABAergic neurons are critical for sensing and regulating hunger signals, including AgRP and POMC neurons. Recent studies highlight their role in modulating feeding behavior beyond classical functions, suggesting a nuanced influence on energy balance. This section delineates the GABAergic nuclei involved in these processes:

### 3.1 Arcuate nucleus: sensing and regulation of hunger signals

In the ARC, GABAergic neurons predominantly fall into two categories:

NPY/AgRP Neurons: commonly referred to as AgRP neurons, these cells synthesize and secrete Neuropeptide Y (NPY) and AgRP. Notably, AgRP neurons are primarily GABAergic in mammals (24). POMC/CART Neurons: often called POMC neurons, they synthesize and secrete POMC. The proportion of GABAergic neurons among POMC neurons varies across species. For example, in rats, approximately 37% of POMC neurons are GABAergic, whereas in mice, this figure rises to about 54%. This variability underscores the diverse function and regulatory mechanism of GABAergic neurons within the ARC across different species (24). Further studies, such as triple *in situ* hybridization (ISH) of the *C57BL/6J* mouse hypothalamus, reveal that within the ARC, 35% of POMC neurons exclusively express Gad67. Moreover, 21% express Vglut2, signifying glutamatergic properties, and 38% co-express both Gad67 and Vglut2, suggesting a subset of POMC neurons capable of both inhibitory and excitatory neurotransmission (25). The distribution and functional diversity of GABAergic neurons within POMC neurons reveals a species-specific adaptation to metabolic and physiological demands, reflecting the intricate regulatory roles of GABAergic neurons in metabolic processes.

#### 3.1.1 Role of GABAergic neurons in the ARC

GABAergic neurons in the ARC play a crucial role in regulating feeding, energy expenditure, and body weight.

##### 3.1.1.1 AgRP neurons

Historically, AgRP/NPY neurons in the hypothalamic arcuate nucleus have been considered essential for maintaining normal feeding and body weight, with their ablation often associated with severe anorexia and reduced body weight (26, 27). Recent studies have highlighted the importance of the Cav3.2 calcium channel in the function of ARC GABAergic neurons. The specific knockout of Cav3.2 reduces neuronal excitability, resulting in decreased food intake, increased energy expenditure, and lower body weight. Notably, naringenin, a bioactive compound found in fruits and herbs, targets Cav3.2, reducing the firing activity of ARC GABAergic neurons and further diminishing food intake and body weight (28). However, a recent study challenges this view, demonstrating that nearly complete ablation of arcuate AgRP/NPY neurons in adult mice did not lead to significant changes in ad libitum feeding or body weight. While the acute inhibition of AgRP neurons reduces short-term food intake, the long-term absence of these neurons does not disrupt normal feeding behavior under standard conditions (29). Notably, the study found that the loss of AgRP/NPY neurons blunted the refeeding response after fasting, highlighting a specific role in adaptive feeding during energy deficits (29). Recent findings further refined our understanding of the role of AgRP neurons in feeding regulation. These neurons are activated in states of hunger or when food is available. Inhibition of AgRP neurons in hungry mice leads to increased exploratory behavior and reduced food intake, while their activation in the presence of food decreases exploratory actions and promotes feeding (30). These findings suggest that, although AgRP neurons are crucial for responding to fasting, they may not be required for the maintenance of body weight during regular, unrestricted feeding, indicating potential compensatory mechanisms within the hypothalamic circuitry.

Interestingly, the role of GABA signaling has been linked to anti-hunger mechanisms. Administration of the exogenous GABA_A_ receptor agonist, bretazenil, can counteract the anorexic effects caused by the absence of AgRP neurons and restore normal feeding behavior in mice (27). Several signals can activate AgRP neurons, affecting feeding behavior and energy metabolism: higher plasma levels of uridine diphosphate (UDP) in obese patients stimulate AgRP neurons via the purinergic receptor P2Y6, enhancing feeding behaviors (31). Elevated O-linked β-N-acetylglucosamine Transferase (OGT) levels during hunger increase AgRP neuron excitability, reducing thermogenesis and energy expenditure to maintain energy reserves. Conversely, knocking out the OGT gene in AgRP neurons reduces their excitability, promotes browning of white adipose tissue, and mitigates diet-induced obesity and insulin resistance (32). Mitofusins 1 and 2 (Mfn1/2) facilitate mitochondrial fusion in AgRP neurons, linking their function to obesity. Post-fasting refeeding reduces mitochondrial number and increases size within AgRP neurons, enhancing mitochondrial fusion. Knockout of Mfn1 or Mfn2 genes disrupt ATP synthesis and breakdown, decrease intracellular ATP levels, reduce AgRP neuronal firing rate, and lead to reduced food intake and obesity (33). Forkhead box transcription factor O1 (FoxO1) sustains the pro-feeding function of AgRP neurons. Knockout of FoxO1 gene in these neurons reduces feeding, increases leptin and insulin sensitivity, and decreases body fat (34). These insights provide a foundation for potential therapeutic interventions aimed at modulating feeding behavior and metabolic health by regulating AgRP neurons.

##### 3.1.1.2 POMC neurons

POMC (pro-opiomelanocortin) neurons in the hypothalamic arcuate nucleus are central to the regulation of appetite and metabolic homeostasis. Ablation of these neurons leads to severe obesity, highlighting their critical role in maintaining energy balance. Interestingly, re-expression of POMC in GABAergic-POMC neurons in Pomc-deficient mouse line (*arcPomc^-/-^*) via tamoxifen induction significantly reduces body weight, normalizes food intake and blood glucose levels, and alleviates hyperphagic behavior following fasting. These results highlight the essential regulatory role of GABAergic POMC neurons, particularly through mechanisms such as mTORC1 signaling (35). Notably, inhibition of mTORC1 in these neurons activates them, promoting food intake and counteracting the anorexigenic effects of glutamatergic POMC neurons, indicating a complex interplay between excitatory and inhibitory POMC subpopulations (36).

However, a recent finding challenge the traditional view that activation of POMC neurons consistently leads to weight reduction. This study, employing multiple animal models, demonstrated that while chronic inhibition of POMC neurons or paraventricular MC4R-expressing neurons induced significant obesity, chronic activation of these neuronal populations failed to reduce body weight. Furthermore, enhancing melanocortin signaling through overexpression of MC4R, POMC, or its derived peptides showed limited effects on obesity prevention or reversal. These results suggest a functional bias in melanocortin action towards protecting against weight loss rather than promoting weight reduction, and provides new insight into the neural basis for the difficulty in achieving effective obesity treatment through POMC or MC4R activation, despite their well-established role in regulating energy balance.

Liraglutide, a pharmacological agonist of the glucagon-like peptide-1 receptor (GLP-1R), is widely used as an effective agent in metabolic regulation. Liraglutide exerts direct effects on POMC neurons in the arcuate nucleus via activation of the TrpC5 channel, enhancing neuronal activity and resulting in appetite suppression and weight loss. In addition, liraglutide influences AgRP neurons by facilitating GABA release at presynaptic terminals, amplifying satiety signals and reducing food intake through the inhibition of AgRP neuron activity (37). This dual modulation of both POMC and AgRP neurons exemplifies a comprehensive approach to managing metabolic health, targeting both appetite suppression and energy expenditure enhancement.

Insulin and leptin synergistically act on hypothalamic POMC GABAergic neurons to promote the browning of white adipose tissue (WAT), thereby facilitating weight reduction and improving metabolic profiles. Enhancing insulin and leptin signaling through the deletion of specific phosphatases like PTP1B and TCPTP in these neurons prevents diet-induced obesity by increasing energy expenditure and stimulating the browning of WAT. Central interventions, such as co-administration of insulin and leptin or direct activation of POMC neurons, have been shown to promote adipose tissue browning, reduce body fat, and enhance overall metabolic health (38). Recent studies have revealed that POMC neurons are functionally heterogeneous, comprising distinct subpopulations that release GABA, glutamate, or both—each exerting different effects on energy balance. In this context, fluoxetine (FLX) appears to exert its anti-obesity effects by selectively remodeling the synaptic inputs on POMC neurons. Specifically, FLX increases AMPA receptor-mediated excitatory transmission while reducing presynaptic GABAergic inhibitory inputs. This shift in the balance of excitatory and inhibitory signals likely enhances the activity of the anorexigenic (appetite-suppressing) POMC subpopulation, thereby promoting weight loss. These findings underscore the importance of synaptic remodeling—particularly the modulation of GABAergic inputs—in FLX’s mechanism of reducing body weight (39). The expression of GABAergic markers in POMC neurons is sensitive to nutritional status. Short-term fasting and prolonged caloric restriction significantly decrease Gad-67 mRNA expression in POMC neurons, whereas Gad2 expression is less affected. Notably, neither acute nor chronic intermittent restraint stress, nor maintenance on a high-fat diet, alters Gad-67 expression, highlighting the specific sensitivity of GABAergic POMC neurons to energy deficits (40, 41). This adaptive response suggests that GABAergic signaling in POMC neurons may play a role in fine-tuning energy balance during states of caloric insufficiency.

In conclusion, POMC GABAergic neurons represent a crucial node in the neural network governing energy balance. Detailed mapping of their synaptic inputs and outputs, combined with a deeper understanding of their molecular signaling pathways, could provide new targets for interventions in obesity, diabetes, and other metabolic disorders.

#### 3.1.2 The Role of non-AgRP and non-POMC GABAergic neurons in the arcuate nucleus

It is well established that the arcuate nucleus (ARC) contains diverse populations of GABAergic neurons beyond the traditionally characterized POMC and AgRP subtypes. Indeed, multiple reports have identified other GABAergic neuronal populations, including prepronociceptin (Pnoc), rat insulin II promoter-Cre (RIP-Cre), and cellular retinoic acid binding protein 1 (Crabp1), highlighting the complexity and heterogeneity of the ARC’s neural architecture. GABAergic rat insulin-2 promoter (*RIP*)-Cre neurons play a role in metabolic regulation. In the *RIP-Cre::Vgat^flox/flox^* mouse model, despite unchanged food intake, these mice exhibit increased body weight and fat mass and decreased oxygen consumption. Activation of RIP-positive GABAergic neurons enhances energy expenditure and thermogenesis in brown adipose tissue, countering diet-induced obesity (42). Further, a distinct class of GABAergic neurons, identified in 2020 as PNOC-expressing neurons, diverges from the typical feeding regulatory pathways of AgRP or POMC neurons. These neurons, are widely distributed within the ARC, provide inhibitory synaptic inputs to anorexigenic POMC neurons; and their activation promotes feeding. Selective ablation of these neurons enhances POMC neuron activation under high-fat diet conditions, reduces feeding, and protects against obesity without affecting normal chow consumption (43).

Recent research has demonstrated that long-term activation of ARC AgRP neurons or hypothalamic GABAergic neurons, by specific expression of the Na^+^ channel NachBac, induces severe obesity and hyperphagia, akin to the phenotypes of leptin-deficient (*ob/ob*) mice. The degree of obesity is independent of the number of activated neurons, indicating redundant mechanisms controlling appetite in the arcuate nucleus. Notably, these phenotypes persist in *ob/ob* mice with specific activation of non-AgRP GABA neurons of the arcuate nucleus and also in those with ablated AgRP neurons. Moreover, leptin treatment does not mitigate the enhanced feeding and obesity in these mice, suggesting that the body weight regulation by ARC GABA neurons and the anti-obesity effects of leptin are not solely dependent on AgRP neurons (44).

Recently, a novel GABAergic population termed Crabp1 neurons was identified in ARC. These neurons express both leptin receptor (LepR) and GLP-1R. Their activation promotes feeding, while nutritional state and GLP-1R agonism inhibit their activity, thereby suppresses appetite (45). This discovery highlights the diverse roles of ARC GABAergic neurons and underscores their roles for the regulatory network of energy balance. Overall, these studies illuminate the important function of GABAergic neurons in the arcuate nucleus, not only in the regulation of appetite and energy balance but also in broader metabolic processes, thus providing potential therapeutic targets for metabolic disorders.

GABAergic neurons in the ARC exhibit context-dependent roles influenced by the metabolic state, acting as key regulators of feeding behavior and energy homeostasis. Future research should focus on elucidating the interactions between GABAergic signaling and other neurotransmitters, such as NPY and AgRP, particularly under varying metabolic and environmental conditions. This will enhance our understanding of the integrative role of ARC GABAergic neurons in complex neural circuits and pave the way for innovative therapeutic strategies targeting appetite regulation and eating disorders.

These lines of evidence suggest that the diversity of GABAergic neurons in hypothalamic regions, including the ARC, extends beyond the simple POMC and AgRP classification, underscoring the need for a more nuanced understanding of their roles in feeding and metabolic regulation. Additionally, future investigations should apply single-cell sequencing and circuit-tracing techniques to map the functional diversity and specific neural connections of GABAergic neurons within the ARC. This comprehensive approach will provide critical insights into the heterogeneity of these neurons and help identify novel intervention targets for precise modulation of feeding behavior and metabolic health.

### 3.2 Paraventricular nucleus: integration of energy status and stress responses

The PVH, located in the supraoptic region adjacent to the third ventricle, serves as an integrative center that receives neural inputs from diverse brain regions—including the bed nucleus of the stria terminalis (BNST), medial preoptic area (MPA), DMH, arcuate ARC, and supraoptic nucleus (SON) (46, 47). This extensive network enables the PVH to coordinate metabolic signals from peripheral tissues such as the gastrointestinal tract and adipose tissue.

The PVH contains very few GABAergic neurons (48). However, some studies have suggested that GABAergic signaling within the PVH may modulate certain aspects of metabolic regulation. For instance, endogenous cannabinoid 2-arachidonoylglycerol (2-AG) has been reported to influence appetite and energy expenditure by inhibiting GABAergic inputs, which in turn can affect the activity of melanocortin 4 receptor (MC4R) neurons during energy-deficit states (49). Similarly, serotonin signaling in the PVH appears to involve GABAergic interneurons in modulating corticotropin-releasing factor (CRF)-producing neurons (50). Under acute stress conditions, changes in the activity of these interneurons may impact the hypothalamic–pituitary–adrenal (HPA) axis and contribute to variations in both metabolic and cardiovascular functions (51, 52).

Additional evidence indicates that GABAergic projections to the PVH, originating from regions such as the LH and the ARC (including inputs from AgRP and tyrosine hydroxylase-positive neurons), may play a role in modulating feeding behavior and circadian metabolic rhythms (53–55). However, it is important to note that the overall contribution of GABAergic neurons in the PVH to the direct regulation of energy homeostasis remains to be fully established. Their influence is likely to be modulatory rather than central, acting in concert with other neurotransmitter systems and neuropeptides.

Future research employing techniques such as single-nucleus RNA sequencing (snRNA-Seq) and cell-type–specific tracing will be critical for delineating the molecular and functional heterogeneity of PVH neuronal populations. Such studies should help clarify the specific roles of GABAergic signaling in the PVH and its interactions with other pathways in regulating metabolic processes.

### 3.3 Dorsomedial hypothalamus: regulation of thermogenesis and energy expenditure

The dorsomedial hypothalamus (DMH), located in the tuberal region of the hypothalamus, is a key center for controlling thermogenesis and maintaining energy balance. The DMH contains abundant GABAergic neuron. The DMH also contains prolactin-releasing peptide (PrRP) neurons, which respond to leptin signaling. Deletion of the leptin receptor (lepR) in PrRP neurons diminishes leptin’s thermogenic effects and promotes obesity, indicating a significant role for these neurons (56).

DMH neurons expressing the TrkB receptor, a receptor for brain-derived neurotrophic factor (BDNF), are crucial for feeding regulation. Chemogenetic activation of these neurons—which include both glutamatergic and GABAergic populations—reduces food intake during the dark cycle, when mice typically exhibit increased hunger. Conversely, inhibition of these neurons during the light cycle (a period of natural satiation) enhances feeding without affecting nocturnal behaviors, indicating a circadian-dependent regulatory role (57). The RIIβ subunit of Protein Kinase A (PKA) is another critical factor in energy homeostasis mediated by DMH GABAergic neurons. *RIIβ* knockout mice exhibit pronounced browning of white adipose tissue (WAT). Interestingly, re-expression of RIIβ in DMH GABAergic neurons reverses this effect, demonstrating that activating these neurons or inhibiting PKA induces WAT browning and reduces body weight (58). This suggests that the RIIβ-PKA signaling pathway in DMH GABAergic neurons is a pivotal regulator of adipose tissue metabolism, and targeting this pathway could offer a novel therapeutic strategy for promoting WAT browning and combating obesity.

Research led by Martin G. Myers Jr. identified a significant population of GABAergic LepRb neurons in the DMH using single-nucleus RNA sequencing (snRNA-Seq), termed LepRbGlp1r neurons. These neurons play essential roles in regulating food intake and body weight. Knockout of leptin receptors in LepRbGlp1r neurons leads to hyperphagia and obesity without altering energy expenditure. Remarkably, restoring Glp1r expression in these neurons in *Glp1r*-deficient mice reinstates the anorexigenic effects of the GLP-1R agonist liraglutide, highlighting the significance of these neurons in mediating the effects of GLP-1 signaling on appetite control (59).

The DMH serves as a critical hypothalamic hub for integrating metabolic and thermoregulatory signals, with GABAergic neurons playing multifaceted roles in modulating energy expenditure, adipose tissue metabolism, and feeding behaviors. Future research should focus on the molecular and functional heterogeneity of DMH GABAergic neurons, employing advanced techniques such as optogenetics, chemogenetics, and single-cell transcriptomics. These approaches can help delineate distinct subpopulations of GABAergic neurons and their specific contributions to metabolic control. Moreover, exploring the interplay between RIIβ-PKA signaling and TrkB receptor pathways in DMH neurons may yield new insights into the regulation of adipose tissue metabolism and provide novel therapeutic avenues for enhancing WAT browning and improving metabolic health.

### 3.4 Ventromedial hypothalamic nucleus: regulation of metabolism and glucose homeostasis

The ventromedial hypothalamus (VMH), located in the tuberal region of the hypothalamus, serves as a critical hub for energy regulation and metabolic homeostasis. Neurons within the VMH express receptors for key metabolic ligands such as leptin, neuropeptide Y (NPY), melanocortin, and cholecystokinin, positioning the VMH as a central integrator of metabolic signals. Notably, GABAergic neurons in the VMH are highly responsive to stress stimuli and play a pivotal role in regulating metabolic balance and glucose homeostasis.

During hypoglycemic states, the synthesis of GABA within the VMH is significantly upregulated, potentially activating physiological mechanisms that increase circulating glucose levels to counteract hypoglycemia (60). However, excessive activation of GABAergic neurons can lead to heightened GABA release, which reduces the excitability of steroidogenic factor 1-expressing (SF1) neurons. This suppression of SF1 neuronal activity impairs glucose and lipid metabolism of females, underscoring a delicate balance between GABAergic inhibition and excitatory signaling in the VMH (61). Recurrent hypoglycemic episodes can amplify GABAergic output from the VMH, leading to counterregulatory failure, where the body’s ability to restore normal glucose levels during subsequent hypoglycemic challenges is compromised (62). These observations emphasize the crucial role of VMH GABAergic neurons in managing glucose homeostasis and responding adaptively to metabolic stress.

Activation of VMH GABAergic neurons under stress conditions can induce anxiety-like behaviors and negatively impact bone health, contributing to bone loss (63). This highlights the broader physiological influence of these neurons and their potential role in stress-related comorbidities.

A novel layer of metabolic regulation involves the lactate receptor GPR81, which differentially affects GABAergic neurons in the anterior and posterior sections of the ventrolateral ventromedial nucleus (VMHvl). Activation of GPR81 in the anterior VMHvl enhances the expression of glutamic acid decarboxylase (GAD), increasing GABA synthesis and release. Conversely, in the posterior VMHvl, GPR81 activation suppresses GAD expression, reducing GABA synthesis (64). This bi-directional modulation by GPR81 suggests distinct regulatory roles for GABAergic neurons along the anterior-posterior axis of the VMHvl, potentially influencing glucose regulation in a spatially specific manner.

Additionally, two isoforms of glycogen phosphorylase in the VMH, GP-muscle (GPmm, responsive to norepinephrine) and GP-brain (GPbb, sensitive to AMP), regulate GABA production in the rostral and caudal regions of the VMH (65). This differential regulation of GABAergic signaling plays a critical role in maintaining glucose levels, particularly during hypoglycemic states. Furthermore, the astrocytic octadecaneuropeptide (ODN) modulates metabolic processes in the VMH through its effects on GABAergic neurons, illustrating the complex neurochemical interactions that govern energy balance within this nucleus (66).

In conclusion, VMH GABAergic neurons represent a central node in the hypothalamic control of energy and glucose metabolism. Understanding their complex regulatory mechanisms and interactions with various metabolic signals will be crucial for developing precision therapies aimed at restoring metabolic balance and improving health outcomes in individuals with metabolic disorders.

### 3.5 Lateral hypothalamic area: regulation of feeding behavior and reward integration

Lateral hypothalamic (LH) GABAergic neurons are pivotal in balancing homeostatic and hedonic drives, orchestrating consummatory behaviors across a diverse array of functional subtypes. Single-cell sequencing has revealed at least 15 distinct GABAergic populations within the LH, underscoring the heterogeneity and complexity of this region (67). These neurons play a key role in stimulating feeding behaviors and are highly responsive to palatable substances like sucrose. Interestingly, head-fixed two-photon microscopy has shown that exposure to high-fat, high-sugar (HFHS) diets reduced the overall responsiveness of these neurons to water and sucrose, while their response to sucrose alone remains elevated (68). This discrepancy suggests that no single subgroup of LH GABAergic neurons exclusively drives these behaviors; instead, it points to the integrative function of the broader GABAergic network.

The ablation of LH GABAergic neurons impairs appetitive learning and reduces weight gain, highlighting their role in regulating dietary behaviors and adaptive feeding responses (69). Activation of LH GABAergic neurons reliably initiates feeding behaviors, such as gnawing, in mice, whereas their inhibition prevents these actions even in hungry animals (29).

Neurotensin (Nts) neurons in the LH, which are GABAergic, play coordinated roles in regulating feeding and drinking behaviors. A subtype of these neurons such as GABA NtsLepRb neurons, which respond to leptin and project to the ventral tegmental area (VTA) and substantia nigra pars compacta (SNc), influences feeding. On the other hand, GABA NtsDehy neurons respond to dehydration signals and regulate drinking behavior without projecting to the VTA or SNc (70). Additionally, recent studies have identified GABAergic projections from the LH to the ventral IPAG (vIPAG), which play a role in regulating satiety. Activation of this pathway induces feeding in satiated mice by inhibiting GABAergic neurons within the vIPAG, demonstrating a complex interplay between hypothalamic and brainstem circuits (71). Moreover, GABAergic inputs from the olfactory cortex influence olfactory-guided feeding behavior, highlighting the diverse sensory integration within the LH (72).

Future research should focus on identifying the functional roles of specific GABAergic subpopulations using advanced tools like spatial transcriptomics, optogenetics, and single-cell calcium imaging. Understanding the context-dependent activity of subtypes such as GERNs and Nts neurons will provide insight into how these circuits adapt under different metabolic and pathological states. Investigating the interactions between LH GABAergic neurons and other hypothalamic and midbrain networks (e.g., orexin and reward pathways) will be critical for developing targeted therapies for obesity and eating disorders, leveraging the precise modulation of these neural circuits to restore balanced feeding behavior.

### 3.6 Hypothalamic tuberal nucleus: control of hunger and feeding behavior

GABAergic neurons in the hypothalamic tuberal nucleus (HTN) play a crucial role in modulating feeding behaviors and integrating hunger signals. Activation of somatostatin (SST) neurons within the HTN significantly influences feeding dynamics. Rodent studies show that the activation of SST GABAergic neurons within the HTN by ghrelin correlates with a robust increase in food consumption, underscoring their direct role in appetite stimulation. Moreover, human clinical data support this finding, as damage to the lateral tuberal nucleus is linked to hyperphagia and excessive appetite, emphasizing the importance of this region in appetite control (73). This connection reflects the critical role of the tuberal nucleus in managing hunger signals and feeding behavior, providing valuable insights into the neural mechanisms underlying appetite regulation.

### 3.7 Hypothalamic preoptic area: thermoregulation and metabolic homeostasis

GABAergic neurons in the preoptic area (POA) of the hypothalamus are key regulators of metabolic and thermoregulatory processes. These neurons play an essential role in the body’s response to thermal stress. For example, a GABAergic neuronal group in the POA that expresses the prostaglandin EP3 receptor plays a pivotal role in thermoregulation by bidirectionally controlling body temperature and mediating fever responses. These neurons send GABAergic projections to the DMH, modulating sympathetic output to brown adipose tissue (BAT) and thereby regulating body temperature and metabolism (74).

### 3.8 Suprachiasmatic nucleus: circadian regulation of metabolism and energy balance

GABAergic neurons in the suprachiasmatic nucleus (SCN) of the hypothalamus are fundamental regulators of circadian rhythms, which are intricately linked to metabolic processes. These neurons are distinguished by their high expression of the nuclear receptors REV-ERB-α and REV-ERB-β, key components of the molecular clockwork. The expression of REV-ERB in SCN GABAergic neurons exhibits strong circadian patterns, peaking just before the wake phase. Glucose clamp studies have demonstrated that control mice display clear circadian variations in insulin sensitivity, hepatic glucose production, and gluconeogenesis, all of which peak during wakefulness. Chemogenetic stimulation of SCN GABAergic neurons during wakefulness induces glucose intolerance. However, restoring the temporal pattern of either SCN GABAergic neuron firing or REV-ERB expression effectively rescues the time-dependent glucose metabolic disruptions caused by REV-ERB depletion. The exacerbated glucose intolerance in *REV-ERB* KO mice is associated with increased firing activity of SCN GABAergic neurons (75). This heightened neuronal activity likely enhances presynaptic release probability, leading to an increase in the frequency and amplitude of miniature excitatory postsynaptic currents (mEPSCs) in downstream target neurons. Deletion of REV-ERB disrupts the rhythmic expression of critical genes, such as Rgs16 and Takusan family members, which are key regulators of synaptic function and glucose homeostasis during the active phase.

These findings illustrate the comprehensive role of SCN GABAergic neurons not only in temporal regulation but also in metabolic processes, with significant implications for understanding and potentially treating metabolic disorders such as diabetes. Developing pharmacological agents that enhance REV-ERB function or mimic its rhythmic activity might offer a new avenue for aligning disrupted circadian and metabolic cycles, with significant potential for clinical applications in metabolic health management.

## 4 Hypothalamic GABAergic pathways: key circuits in metabolism and energy regulation

**Figure 2:**
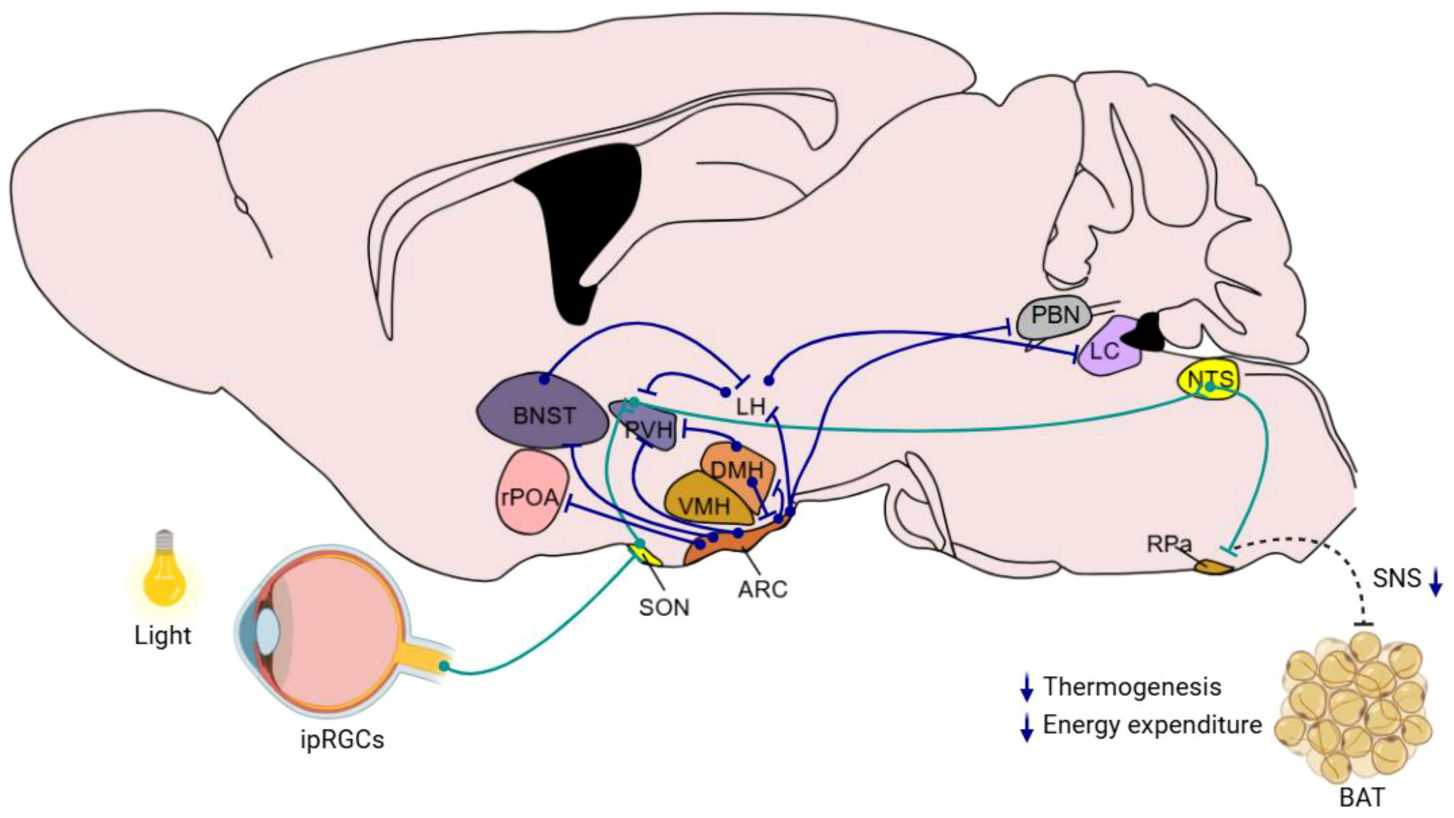
Hypothalamic GABAergic nuclei and neural pathways involved in metabolism and energy balance regulation This schematic diagram illustrates the organization of key hypothalamic GABAergic nuclei, such as the ARC, DMH, and other relevant regions, and their interconnections with neural circuits implicated in metabolic control. Blue lines indicate the direct direction of neuronal projections that modulate functions including appetite regulation, energy expenditure, and glucose homeostasis. Green lines indicate the indirect neural chain from retina to RPa. The diagram integrates current insights into how these GABAergic pathways interact with peripheral systems to maintain energy balance.

The hypothalamus is a central hub in regulating metabolic homeostasis and energy balance, which are critically influenced by its GABAergic neuronal system. This system not only operates within the hypothalamus but also coordinates with broader neural networks including the brainstem and limbic system. This overview outlines the GABAergic neuronal projections both within the hypothalamus and the extending areas to other brain regions, emphasizing their roles in metabolic regulation (**Figure 2**). Here is a summary of the hypothalamus-originated metabolic regulation-related neural projections:

### 4.1 Arcuate nucleus to paraventricular nucleus

The neural pathways connecting the ARC and the PVH of the hypothalamus are fundamental in maintaining metabolic homeostasis. AgRP neuronal fibers and glutamatergic neuronal fibers from the ARC converge on the PVH. In addition, GABAergic neuronal terminal, from the ARC to melanocortin-4 receptor (MC4R) neurons in the PVH, releases AgRP to antagonize MC4R and thus promotes feeding. This interaction illustrates the complex interplay of different neurotransmitters in regulating energy balance. Additionally, fasting activates ARC AgRP neurons, which subsequently trigger the hypothalamic-pituitary-adrenal (HPA) axis. AgRP neurons facilitate this by reducing GABAergic input to PVH corticotropin-releasing hormone (CRH) neurons through presynaptic inhibition at BNST neuronal terminals within the PVH. This disinhibition of PVH CRH neurons leads to HPA axis activation, elevating blood glucocorticoid levels and consequently increasing blood glucose (51).

The ARC also features a specific projection involving RIP-positive GABAergic neurons that extend to the PVH and further to the nucleus of the solitary tract (NTS). Activation of this projection enhances energy expenditure by releasing GABA to downregulate excitatory neurotransmission from the PVH to the NTS, increasing thermogenesis in brown adipose tissue and elevating basal metabolic rate without affecting food intake (42). Furthermore, some of ARC POMC neurons can send GABAergic projections directly to the PVH, targeting melanocortin-4 receptor (MC4R)-expressing satiety neurons and inhibiting their activity. Activation of these GABAergic inputs promptly inhibits PVH satiety neurons, thereby triggering immediate feeding behavior (76).

Another significant pathway involves POMC GABAergic neurons from the ARC projecting to the PVH. Blocking the mTORC1 signal in these neurons activates them, thereby strengthening the GABAergic projection from ARC POMC neurons to the PVH. This activation promotes the release of endocannabinoid anandamide (AEA) in the PVH, leading to an increase in food intake (36). These pathways demonstrate the nuanced neurobiological mechanisms involved in regulating energy balance and food intake, highlighting a sophisticated network of metabolic control within the hypothalamus.

### 4.2 Arcuate nucleus to dorsomedial hypothalamus

GABAergic neurons in the AgRP-DMH neural circuit are involved in controlling leptin-mediated suppression of feeding and weight management. Within this pathway, AgRP neurons that express the leptin receptor (LepR) send GABAergic projections to the dorsomedial hypothalamic nucleus (DMH), specifically targeting neurons that express the α3-GABA_A_ receptor (77). Research has shown that acute activation of this GABAergic AgRP-DMH circuit leads to increased food intake and worsened glucose intolerance, while activating MC4R neurons in the DMH has the opposite effects (77). This demonstrates the circuit’s bidirectional role in dietary and energy balance regulation. Moreover, swiftly eliminating LepR from AgRP neurons induces obesity, whereas inhibiting GABA_A_ receptors in the DMH can reverse this obesity phenotype (77). Moreover, the DMH is a primary target for GABAergic neurons expressing pro-opiomelanocortin (POMC). Research indicates that approximately 75% of ARC-POMC neurons projecting to the DMH are GABAergic. Activation of these ARC-POMC GABAergic neurons inhibits neuropeptide Y (NPY) levels in DMH neurons, which suppresses appetite (35). These observations indicate that DMH GABAergic neurons influence leptin’s regulatory effects on appetite suppression and energy balance, providing new perspectives on leptin’s function in energy homeostasis and its potential therapeutic applications for obesity.

### 4.3 Arcuate nucleus to parabrachial nucleus

AgRP neurons in the ARC which co-express AgRP, NPY, and GABA, play a critical role in promoting feeding. Studies have shown that ablation of these neurons in adult mice leads to increased Fos activation in postsynaptic neurons and subsequent starvation. This starvation phenotype is linked to the loss of GABAergic signaling, as chronic administration of bretazenil—a partial GABA_A_ receptor agonist—suppresses Fos activation and maintains feeding during AgRP neuron ablation. Furthermore, direct delivery of bretazenil into the parabrachial nucleus (PBN), a key downstream target of AgRP neurons involved in relaying gustatory and visceral sensory information, is sufficient to sustain feeding. Conversely, inhibition of GABA synthesis in the ARC or blockade of GABA_A_ receptors in the PBN induces anorexia. These findings highlight the essential role of ARC to PBN GABAergic projections in modulating feeding behavior (27).

### 4.4 Arcuate nucleus to bed nucleus of the stria terminalis

PNOC neurons, which are GABAergic and broadly distributed in the ARC, play roles in feeding regulation. Optogenetic activation of the ARC PNOC neurons to BNST projection enhances feeding behavior, while selective ablation of these neurons decreases feeding and protects against obesity (43).

### 4.5 Arcuate nucleus to lateral hypothalamus

The projections from the ARC POMC neurons to the LH are important in controlling feeding behavior. It was well known that leptin-responsive GABAergic neurons are located in the ARC, the DMH, and the LH (55), and leptin could reduce IPSC frequency in POMC neurons by 25% in one-third of POMC neurons (78). Further study show that Leptin may target predominantly GABAergic ARC POMC neurons (55), prompting the release of melanocortins into the lateral hypothalamus (LH), thereby reducing food intake.

### 4.6 Arcuate nucleus to rostral preoptic area

Leptin receptor-positive GABAergic neurons in the ARC extend projections to neurons in the rostral preoptic area (rPOA), establishing a vital link in the hypothalamic-pituitary-gonadal axis. Research has shown that approximately 10-45% of leptin-responsive GABAergic neurons have the capability to project to the rPOA (79). This connection is instrumental in managing both reproductive and metabolic functions, suggesting the integrated role of these neurons in coordinating physiological processes.

### 4.7 Bed nucleus of the stria terminalis to lateral hypothalamus

The endocannabinoid system within the BNST impacts GABAergic neurons in the LH through CB1 receptors, thereby modulating the cardiovascular response to stress. Activation of the BNST by stress leads to an enhancement of GABAergic neurotransmission to the LH via CB1 receptors. This increased GABAergic signaling reduces the expression of Fos protein in the LH, helping to regulate cardiovascular responses and specifically mitigating tachycardia and elevated metabolism associated with restraint stress. Application of the CB1 receptor antagonist AM251 in the BNST blocks this regulatory process, resulting in intensified stress-induced tachycardia. Moreover, pre-treatment with the GABA_A_ receptor antagonist SR95531 in the LH abolishes the effects of AM251 on tachycardia, further demonstrating the role of BNST GABAergic neurons in mediating stress responses through BNST CB1 receptors (80).

### 4.8 Lateral hypothalamus to paraventricular nucleus

GABAergic inhibitory projections from the LH to the PVH play a role in the regulation of feeding. LH GABAergic neurons project extensively to the PVH. Optogenetic activation of this pathway stimulates GABA release, which induces monosynaptic inhibitory postsynaptic currents (IPSCs) in PVH neurons, effectively promoting feeding behavior (53).

### 4.9 Dorsomedial hypothalamus to arcuate nucleus

The GABAergic projection from the DMH to the ARC plays important role in controlling feeding behavior. During fasting periods, GABAergic neuronal terminals from the DMH demonstrate an increased readily releasable pool of GABA vesicles and a heightened probability of release, which amplifies the inhibition acted on POMC projecting neurons. This intensification of inhibition lowers the excitability of POMC neurons, leading to an increase in food intake. Conversely, during periods of energy surplus, this GABAergic transmission is diminished to disinhibit POMC neurons, increasing their excitability and thereby enhancing satiety signals, which results in decreased food intake (40).

### 4.10 Dorsomedial hypothalamus to paraventricular nucleus

DMH neurons expressing leptin receptors (LepR-positive) also send inhibitory projections to the PVH. Leptin’s action through this pathway reduces the excitability of interscapular BAT (iBAT)-related PVH neurons, playing a role in overall metabolic regulation (81).

### 4.11 Lateral hypothalamus to locus coeruleus

Early studies indicate that GABA neurons in the LH project to ventral tegmental area (VTA), and activation of these projections results in the inhibition of GABAergic neurons within the VTA. This inhibition was believed to enhance dopamine activity and release, a mechanism thought to drive feeding behavior. However, recent findings challenge this understanding, showing that disrupting GABA neurons in the VTA does not affect stimulus-induced feeding. Additionally, direct activation of dopamine neurons in the VTA or substantia nigra does not trigger foraging or feeding behaviors, suggesting the involvement of alternative regulatory pathways. Further investigations have revealed that LH GABA neurons project beyond the VTA, reaching areas near the locus coeruleus (LC). Stimulating these GABAergic fibers or neurons near the LC triggers feeding behaviors similar to those observed when LH neurons are activated. Importantly, destroying these specific GABA neurons near the LC prevents LH-induced feeding behaviors without impacting overall body weight (82). These findings uncovered the role of specific neural circuits involving the LH and nearby LC regions in promoting overeating, potentially leading to obesity. This pathway operates independently from traditional hunger and satiety regulation mechanisms, illustrating the complex network of brain regions involved in the control of feeding behavior.

### 4.12 Indirect neural chain from retina to suprachiasmatic nucleus

Public health studies suggest that artificial light is a significant risk factor for metabolic disorders. However, the neural mechanisms through which light influences metabolic regulation remain unclear. Research has demonstrated that light acutely impairs glucose tolerance in mice through activation of intrinsically photosensitive retinal ganglion cells (ipRGCs) that project to the SON. From there, antidiuretic hormone neurons project to the SCN, where vasopressin neurons extend their projections to the paraventricular nucleus (PVH). The PVH then sends inhibitory projections to GABAergic neurons in the nucleus of the solitary tract (NTS), ultimately affecting BAT. This activation by light directly inhibits adaptive thermogenesis in BAT, thus reducing glucose tolerance. In humans, light also regulates glucose tolerance at temperatures conducive to brown fat activity (83).

## 5 Pharmacological agents modulating metabolism and energy balance via hypothalamic regulation

Hypothalamic GABAergic neurons are intricately involved in metabolic regulation, and their dysfunction leads to various metabolic diseases such as obesity, diabetes, and metabolic syndrome. Here’s how different pathways and mechanisms underlie the above-mentioned processes: Apoptosis of oligodendrocyte precursor cells in the medial eminence of the hypothalamus can induce leptin resistance in ARC GABAergic neurons, leading to obesity (84). Knockout of RIIβ expressed in hypothalamic GABAergic neurons can increase insulin sensitivity, enhance white adipose tissue browning, increase energy expenditure, and resist obesity induced by a high-fat diet. Conversely, re-expression of RIIβ in DMH GABAergic neurons can reverse the white adipose browning phenotype, returning body weight and fat mass to wild-type levels (58). Endocannabinoid 2-arachidonoylglycerol (2-AG) regulates MC4R cells in the PVH by inhibiting incoming GABAergic signals, controlling feeding behavior. Changes in endogenous cannabinoids (eCBs) signaling strength are inversely related to energy state, where impaired synthesis of 2-AG in MC4R neurons leads to weight loss, increased serum leptin sensitivity, reduced appetite, increased energy expenditure, and resistance to diet-induced obesity (49). In the SCN, GABAergic neurons, especially those containing the nuclear receptor REV-ERB, play a critical role in regulating the diurnal rhythm of insulin sensitivity. The expression of REV-ERB in SCN GABA neurons regulates circadian rhythms by modulating the firing activity of SCN neurons and the gene expression related to neurotransmission. Activation of these neurons at specific times (like wakefulness) can reduce glucose tolerance, whereas normalizing REV-ERB expression or SCN GABA neuron firing rhythm can improve glucose metabolism disrupted by REV-ERB deficiency (75). Intranasal administration of the glio-peptide octadecaneuropeptide (ODN) has a gender-dependent differential control over aromatase in GABAergic neurons, confirming its hypoglycemic effect in male patients, dependent on its activating effect on hypothalamic insulin sensitivity (85). Inhibiting the IKKβ/NF-κB signaling pathway in the medial basal astrocytes of the hypothalamus can enhance the plasticity of these astroglial cells. This, in turn, facilitates the rapid clearance of GABA from synaptic clefts via GABA transporters and increases the expression of the BDNF. These changes can subsequently improve glucose intolerance and hypertension induced by nutritional excess. This approach highlights the role of hypothalamic astrocytes in modulating neuronal activity and metabolic responses, providing a potential target for therapeutic interventions in metabolic disorders (86). Moreover, mutations in the AMPK γ2 subunit that activate AMPK can enhance oxidative phosphorylation in neurons of the medial basal part of the hypothalamus. This activation increases the excitability of AgRP neurons and enhances the sensitivity of both the arcuate nucleus and the paraventricular nucleus to ghrelin, a hormone that stimulates hunger. This enhanced neural and hormonal sensitivity promotes hepatic *de novo* lipogenesis, insulin secretion, and increases insulin sensitivity in muscles. Such physiological changes lead to overeating, β-cell dysfunction, obesity, hepatic steatosis, and symptoms of metabolic syndrome (87).

Here is a summary of drugs that modulate metabolism and energy balance through hypothalamic regulation, as outlined in **Table 1**.

**Table 1:** Agents modulating metabolism and energy balance via hypothalamic GABAergic neurons.

Drug/Compound	Target	Mechanism of Action	Targeted Effect	Ref.
Bretazenil	GABA_A_ receptor	↓ anorexic behavior	restored feeding behavior	(28)
Uridine diphosphate (UDP)	Purinergic receptor P2Y6	↑ AgRP neuronal excitability ↑ feeding behavior	increased adiposity	(32)
O-linked β-N-acetylglucosamine Transferase (OGT)	AgRP neurons	↑ AgRP neuronal excitability ↓ thermogenesis ↓ energy expenditure	induced DIO and insulin resistance	(33)
Rapamycin	mTORC1 signaling in POMC neurons	↑ food intake	increased adiposity	(37)
Liraglutide	TrpC5 channel of POMC neurons	↑ insulin secretion ↓ appetite	resistance to DIO	(38, 46)
insulin and leptin	LepR of POMC neurons	↑ leptin sensitivity ↑ browning process ↑ energy expenditure ↓ food intake ↓ body weight	resistance to DIO	(39)
2-arachidonoylglycerol (2-AG)	PVH MC4R	↑ appetite ↓ energy expenditure	increased adiposity	(50)
Neuropeptide Y (NPY)	DMH GABAergic neurons	↑ browning process	resistance to DIO	(56)
Glycogen phosphorylase	VMH GABAergic neurons	↑ glucose levels	maintain blood glucose homeostasis	(66)
Octadecaneuropeptide (ODN)	AMPKα1/Prkaa1 and AMPKα2/Prkaa2 of VMH GABAergic neurons	↓ glucose levels	maintain blood glucose homeostasis	(67)
Muscimol	Preproorexin neurons	↓ orexin ↓ food intake	decreased adiposity	(70)
Anandamide (AEA)	POMC neurons	↑ food intake	increased adiposity	(77)

↑ for Enhancement; ↓ for Inhibition.

## 5 Conclusion and future prospects

The hypothalamus serves as the central hub for the regulation of metabolic homeostasis and energy balance. Neurons within the hypothalamus play pivotal roles in maintaining metabolic homeostasis and energy equilibrium. Dysfunction of the hypothalamic neurons is a critical factor in the development of diseases such as obesity and type 2 diabetes. Investigating the mechanisms of metabolic regulation in the hypothalamus and its roles in related diseases can enhance our understanding of the body’s energy and metabolic regulation mechanisms, as well as the pathogenesis of metabolic disorders. Future research focusing on the hypothalamus will continue to provide new insights and approaches for the prevention and treatment of metabolic-related diseases. Deepening the understanding of hypothalamic functions and dysfunctions can develop more targeted and effective therapeutic strategies that address the underlying causes of these complex conditions. This research not only has the potential to improve treatment options but also to offer preventive measures that could mitigate the impact of metabolic diseases on global health.
